# Risk Factors for Wound Dehiscence After Spinal Metastasis Surgery and a New Approach to Prevention—Curved Skin Incision

**DOI:** 10.3390/cancers17121973

**Published:** 2025-06-13

**Authors:** Kunihiko Miyazaki, Yutaro Kanda, Takashi Yurube, Yoshiki Takeoka, Takeru Tsujimoto, Tomoya Matsuo, Masao Ryu, Naotoshi Kumagai, Kohei Kuroshima, Yoshiaki Hiranaka, Ryosuke Kuroda, Kenichiro Kakutani

**Affiliations:** Department of Orthopedic Surgery, Kobe University Graduate School of Medicine, Kobe 650-0017, Japan

**Keywords:** curved skin incision, midline skin incision, spinal metastasis, wound dehiscence, propensity score matching, radiotherapy

## Abstract

**Background**: Postoperative wound dehiscence is a major complication following spinal metastasis surgery, particularly in patients who receive preoperative radiotherapy or molecular-targeted therapy; however, preventive strategies remain limited. **Objective**: In this study, we aimed to identify the risk factors for postoperative wound dehiscence and evaluate the clinical utility of a novel curved skin incision (CSI) technique, designed to avoid irradiated areas, in comparison with the conventional midline incision (MI) technique. **Methods**: Logistic regression analysis was conducted on 107 patients who underwent MI between 2013 and 2018. Based on the results, we developed the CSI technique. Propensity score matching was performed to compare postoperative wound dehiscence in 29 matched pairs of patients treated with either CSI or MI from 2019 to 2021. **Results**: Preoperative radiotherapy and molecular-targeted therapy were found to be significant risk factors for wound dehiscence. CSI, which circumvents irradiated skin, was associated with a substantially lower rate of wound dehiscence than MI. **Conclusions**: The CSI technique offers a simple, reproducible, and effective surgical approach to reduce postoperative wound complications in high-risk patients. Its clinical benefit, especially for those with prior radiotherapy, suggests that it may serve as a valuable addition to standard spinal metastasis surgery.

## Simple Summary

This study investigated risk factors and preventive measures for postoperative wound dehiscence in spinal metastatic surgeries. Logistic regression identified preoperative radiotherapy and molecular-targeted therapy as significant risk factors. A curved skin incision, designed to avoid irradiated areas, significantly reduced wound dehiscence compared with a traditional midline incision.

## 1. Introduction

In recent years, the advent of molecularly targeted drugs, immune checkpoint inhibitors, and advances in anticancer drugs have improved cancer treatment outcomes, resulting in prolonged life expectancy. Bone is the third most frequent site of metastasis after the lung and liver, with the spine being the most commonly involved skeletal site [1]. Consequently, the number of cancer survivors with bone and spinal metastases has increased [2,3]. Approximately 28.9% of patients with known spinal metastases develop symptomatic spinal metastases (SSM), which significantly reduces patient performance status (PS), activities of daily living (ADL), and quality of life (QOL) [4].

The primary objective of multidisciplinary cancer treatment is to improve and maintain PS, ADL, and QOL until the terminal phase, even in patients with advanced spinal metastases. Radiotherapy remains the mainstay of treatment for patients with localized bone pain, achieving partial or complete relief in 50–80% of cases [5]. However, a single radiotherapy treatment is ineffective for treating neurological deficits or spinal instability. Previous reports have recommended combining spinal surgery with radiation therapy for non-ambulatory patients and patients with intractable pain based on their clinical data regarding PS, ADL, and QOL improvement [4,6,7,8]. Further, recent studies have highlighted the increasing complexity of managing spinal metastases in the era of precision medicine, where patients often receive multiple lines of targeted therapy and immunotherapy [9]. This has led to an increased incidence of treatment-related complications, particularly wound healing disorders following surgical interventions [10,11].

Postoperative complications occur in approximately 20–30% of patients who undergo spinal metastasis surgery [10,11]. Wound dehiscence is one of the most devastating complications, which can lead to infection and regression [11]. The pathophysiology of wound dehiscence in irradiated tissues involves impaired collagen synthesis, reduced angiogenesis, and a compromised immune response, making prevention strategies crucial for optimal patient outcomes [12]. Treating wound dehiscence typically involves negative pressure wound therapy and valvuloplasty. However, it takes 3–6 months to return to expected PS and ADL levels and longer hospitalization despite limited prognosis [8]. Clinically, wound dehiscence initially occurs as small ulcers at the radiotherapy site, gradually dissociates, and develops into a deep infection. Several minimally invasive surgical approaches involving midline or paramedian linear incisions have been explored to reduce postoperative complications [13,14]. Nevertheless, effective preventive measures are yet to be established [12,15]. Therefore, we first identified the risk factors for postoperative wound dehiscence after spinal surgery for spinal metastases at our institution (phase 1). Based on these findings, we devised a curved skin incision (CSI) method to prevent ulceration at the radiotherapy sites. The CSI technique represents a novel approach designed specifically to circumvent irradiated tissue zones, thereby potentially reducing wound healing complications. This technique involves creating an arc-shaped incision that maintains adequate surgical exposure while avoiding areas with compromised healing capacity due to prior radiation therapy [11,12]. This study aimed to validate the effects of CSI on wound dehiscence following spinal surgery.

## 2. Materials and Methods

This study was approved by the Institutional Review Board of Kobe University Hospital, Japan (protocol code: B190002 and date of approval: 16 April 2019) and performed in accordance with the ethical standards as laid down in the 1964 Declaration of Helsinki and its later amendments or comparable ethical standards. Written informed consent was obtained from each patient in accordance with Principle 11 of the Declaration of Helsinki and the applicable laws and regulations of Japan.

Patients who underwent palliative surgery for spinal metastases between 2013 and 2021 were prospectively enrolled in this study. Spinal metastasis was diagnosed by plain radiography, computed tomography, magnetic resonance imaging, bone scintigraphy, positron emission tomography, and histological evaluation of needle biopsy samples. We defined SSM as spinal metastases associated with progressive neurological deficits or intractable pain resistant to conservative care, including opioid use. All the patients completed a 6-month follow-up.

Surgical indications included progressive neurological deficits and intractable pain which was resistant to conservative care, including opioid use. At our institution, open decompression is selected for cases with progressive neurological deficits, radiologic evidence of spinal cord compression, or intractable pain not controlled by conservative management. Percutaneous pedicle screws are typically selected when neural decompression is not required. Surgery was contraindicated in patients with impaired consciousness due to cerebral metastasis. Patients who underwent decompression alone, percutaneous posterior fixation, total en bloc spondylectomy, and anterior or combined approaches were excluded to ensure uniformity. The characteristics listed in Table 1 [including age, sex, body mass index (BMI), Revised Katagiri score [16], smoking history, preoperative radiotherapy, chemotherapy, and molecular targeted drug therapy] were selected based on previous studies, which identified them as potential risk factors for postoperative complications in spinal metastasis surgery. These factors were systematically evaluated in our multivariate and propensity score analyses. First, a multivariate analysis was performed to identify the risk factors for wound dehiscence in patients who underwent spinal surgery with a midline incision (MI) until 2018. Next, consecutive patients who underwent spinal surgery with CSI or MI after 2019 were enrolled for the statistical analysis to evaluate the efficacy of CSI using propensity score matching. The treatment approach for all the patients until 2018 was MI, whereas patients after 2019 received either CSI or MI. The selection of CSI or MI was decided for every three patients; CSI was performed on the patients whose ID was a multiple of three.

### 2.1. Surgical Methods

All the surgeries involved single-stage posterior decompression with posterolateral partial tumor removal and posterior stabilization were performed by senior surgeons. All the immobilization devices and collars were removed postoperatively. All the patients received radiotherapy approximately 2 weeks postoperatively. If indicated, an oncologist administered chemotherapy. All the patients, except those with allergies, received perioperative cefazolin for antibacterial prophylaxis for 2 days.

**Figure 1:**
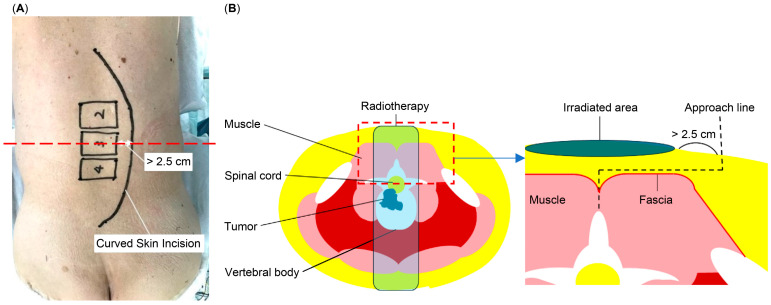
(**A**) Diagram illustrating the curved skin incision technique, indicating the arc-shaped incision placed at least 2.5 cm from the outer edge of the primarily affected vertebra. The red dashed line represents the axial plane used for the cross-sectional schematic shown in (**B**). (**B**) Schematic cross-section depicting the radiotherapy treatment area and the approach for the curved skin incision designed to avoid the irradiated region.

In both the MI and CSI methods, all the patients underwent conventional open surgery, with pedicle screws inserted into three vertebrae caudal and cranial to the metastatic site. Laminectomy was performed based on the patient’s neurological status. Generally, radiotherapy was performed on one vertebra caudal or cranial to the affected vertebrae. The CSI technique was developed as a novel preventive strategy to minimize postoperative wound dehiscence in patients with prior radiotherapy. To avoid irradiated areas with compromised healing potential, an arc-shaped skin incision was made at least 2.5 cm lateral to the outer edge of the affected vertebra. This generally resulted in a skin incision that extended 3–4 cm further laterally compared to conventional MIs according to retrospective intraoperative records. After the incision, subcutaneous fat was carefully mobilized toward the midline without damaging the fascia. Once the spinous process was adequately exposed, the surgery proceeded using standard techniques. This approach preserved full surgical access while reducing the risk of wound complications in irradiated tissue zones (Figure 1).

### 2.2. Study Variables and Outcomes

The patients were followed up for 6 months. Patient data, including age; sex; BMI; Revised Katagiri score; smoking history; preoperative radiotherapy; preoperative chemotherapy; preoperative molecular target drug (MTD) therapy; and treatment-related factors, such as operative time and intraoperative blood loss, were extracted through chart review. Information on the site and origin of spinal metastasis was obtained and recorded. The primary outcome was postoperative wound dehiscence, which was defined as wound separation occurring either with or without signs of surgical site infection. Infectious cases were characterized by purulent discharge, positive wound cultures, or fever, whereas non-infectious cases were clinically diagnosed based on physical findings of wound retraction without evidence of infection. Data on other postoperative complications, such as fracture, rod failure, pneumonia, neurological complications, deep vein thrombosis, and cerebrospinal fluid leak, were also collected. Hematologic malignancies such as multiple myeloma and lymphoma were included in this study, as these conditions, although different in pathophysiology from solid tumors, are clinically relevant to spinal metastasis surgery and frequently require surgical intervention. The inclusion of these cases reflects real-world clinical practice in spinal oncology.

### 2.3. Statistical Analysis

A multivariable logistic regression model was used in the phase 1 cohort to investigate the risk factors for postoperative wound dehiscence. Other factors identified in previous studies to be associated with postoperative wound dehiscence, including age (≥65 years); sex (male); BMI; Revised Katagiri score; smoking history; preoperative radiotherapy; preoperative chemotherapy, preoperative MTD therapy; and treatment-related factors, such as operative time and intraoperative blood loss, were included in the multivariable model. The Mann–Whitney U or Fisher’s exact probability test was used to compare the MI and CSI groups in phase 2, with statistical significance set at *p* < 0.05. To reduce treatment selection bias in comparing MI and CSI, 1:1 propensity score matching, using a caliper size of 0.2, was performed for all the aforementioned factors, and postoperative wound dehiscence was compared between the two groups. A *p*-value less than 0.05 was considered statistically significant. Statistical analyses were performed using the SPSS software (version 11.0; SPSS Inc., Chicago, IL, USA).

## 3. Results

### 3.1. Patient Characteristics

**Figure 2:**
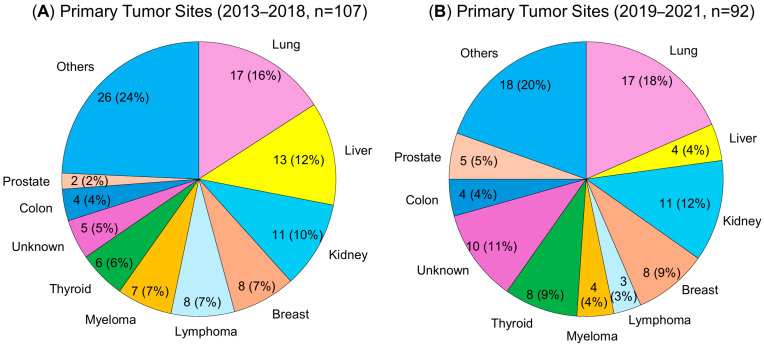
Distribution of primary tumor sites in patients undergoing spinal metastasis surgery: (**A**) patients treated from 2013 to 2018 (midline incision only, MI group). (**B**) patients treated from 2019 to 2021 (midline or curved skin incision, MI or CSI groups).

The demographic data and surgical information of the patients are presented in Table 1. Between 2013 and 2017, 134 patients underwent surgery for spinal metastasis. After excluding 8 cases of decompression, 17 cases of percutaneous posterior fusion, and 2 cases of total tumor resection, 107 cases were included in the study. The patients had a median age of 69 years, were more likely to be male (61%), and approximately half had received preoperative radiotherapy or chemotherapy. The distribution of primary tumor sites between the two cohorts—those treated from 2013 to 2018 and those treated from 2019 to 2021—is shown in Figure 2. Among the primary tumor sites, the lungs were the most common (16%), followed by the liver (12%) and kidneys (10%). This figure supplements Table 1 by visually highlighting the overall consistency in cancer types across both groups.

Similarly, 103 patients underwent surgery for spinal metastases between 2019 and 2021. After excluding 6 cases of decompression and 5 cases of percutaneous posterior fusion, 92 patients (median age, 71 years) were included. Of these, 33 patients underwent CSI and 59 patients underwent conventional MI surgery. Preoperative characteristics were comparable between the patients in the two study periods (Table 1). The distribution of primary tumor sites in the 2019–2021 cohort is illustrated in Figure 2B, showing a composition similar to that of the MI group treated between 2013 and 2018.

### 3.2. Postoperative Wound Dehiscence in Patients with MI (Enrolled in 2013–2017)

**Figure 3:**
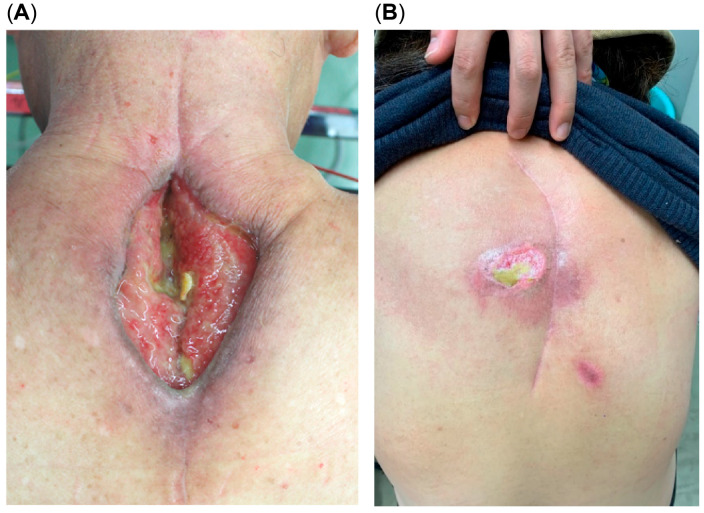
(**A**) Postoperative wound dehiscence following conventional surgery with a midline incision after radiotherapy. (**B**) Postoperative appearance of a case with a curved skin incision showing skin ulceration without wound dehiscence.

Postoperative wound dehiscence was the most common complication in the patients who underwent MI surgery (n = 9 of 107; 8.4%), followed by fracture, pneumonia, rod failure, and deep vein thrombosis (Table 2). All nine patients with postoperative wound dehiscence required revision sutures, three patients required negative pressure wound therapy, and one required valvuloplasty. Postoperative wound dehiscence was more common in male patients (7/9 patients, 77.8%) and in the patients who underwent preoperative radiotherapy (8/9, 88.9%), chemotherapy (7/9, 77.8%), and MTD therapy (6/9, 66.7%). MTD therapies included bevacizumab (n = 2), sunitinib (n = 1), sorafenib (n = 1), sunitinib/sorafenib (n = 1), and sunitinib/temsirolimus (n = 1). Postoperative wound dehiscence occurred in 8 of 31 (25.8%) and 6 of 17 (37.5%) patients who received preoperative radiation and MTD therapies, respectively. The patients receiving preoperative radiation and MTD therapies had a high rate of postoperative wound dehiscence (5 out of 8 cases, 62.5%). Most patients with wound dehiscence developed ulcers at the surgical incision site followed by wound dehiscence (Figure 3A). The drug names and analysis of postoperative wound dehiscence for the 17 cases in which preoperative MTD therapies were administered are shown in Table S1. In the CSI group, one patient developed superficial skin ulceration in the irradiated area, but it did not progress to wound dehiscence. The postoperative appearance of this case is shown in Figure 3B.

The multivariate logistic regression analysis identified preoperative radiation (odds ratio [OR] = 32.599, *p* = 0.004) and MTD therapy (OR = 25.242, *p* = 0.017) as significant risk factors for postoperative wound dehiscence in the patients who underwent MI (Table 3).

### 3.3. Postoperative Wound Dehiscence in Patients with MI or CSI (Enrolled in 2019–2021)

There were no significant differences in the general characteristics of the patients treated with MI or CSI, as shown in Table 4. Preoperative radiotherapy was administered to 22 patients (11 each with MI and CSI) and preoperative MTD therapy was administered to 16 patients (10 with MI and 6 with CSI). Postoperative wound dehiscence was observed in 7 out of 59 (11.9%) patients treated with MI and in 1 out of 33 (3.0%) patients treated with CSI. The drug names and detailed analysis of postoperative wound dehiscence in the 16 patients to whom preoperative MTD therapies were administered are shown in Table S2.

### 3.4. Efficacy of CSI

Propensity score matching was conducted to identify 29 patients in each group. The differences between MI and CSI decreased with propensity score matching. The results before and after the propensity score matching are presented in Table 4. Postoperative wound dehiscence rates were significantly higher in the patients treated with MI (6 of 29 cases [20.7%]) than in those treated with CSI (0 of 29 cases [0.0%]; *p* = 0.024).

## 4. Discussion

In the current study, the multivariate analysis identified preoperative radiation and MTD therapies as the significant risk factors for postoperative wound dehiscence in patients with MI. Further, CSI significantly reduces the risk of postoperative wound dehiscence in patients undergoing spinal metastasis surgery.

The CSI technique offers several distinct advantages over conventional approaches. Most importantly, our study demonstrated a statistically significant reduction in wound dehiscence rates (0.0% vs. 20.7%, *p* = 0.024) when CSI was employed. This technique is particularly valuable because it is simple to implement, requires no specialized equipment, and can be readily adopted by spine surgeons across different healthcare settings. Unlike complex reconstructive procedures or expensive technologies, CSI represents a practical solution that addresses a common and serious complication in spinal metastasis surgery.

Wound dehiscence is one of the major postoperative complications of spinal metastasis, occurring in approximately 4–20% of patients [17], and significantly reduces the QOL of patients. Consistent with our findings, previous studies have shown that postoperative wound dehiscence is associated with several risk factors, including preoperative radiotherapy and chemotherapy, age, steroid use, length of skin incision, previous spinal surgery, and posterior surgical approach [8,17,18]. Previous studies have shown that radiotherapy increases the risk of postoperative wound-related complications, including surgical site infections and wound dehiscence [17,18]. Notably, one study reported a wound complication rate of up to 46% in patients who underwent surgery within 7 days after preoperative radiation therapy, compared to 20% in those with a longer interval between radiation and surgery, which is comparable to the 25.8% dehiscence rate we observed in the patients receiving preoperative radiation [19]. Irradiation suppresses fibroblasts and affects growth factors such as transforming growth factor-beta and vascular endothelial growth factor (VEGF), thereby increasing susceptibility to infectious bacteria and decreasing skin strength [15]. However, evidence regarding the effect of MTD therapy on postoperative wound healing remains limited. VEGF-targeted therapies such as bevacizumab, among other MTDs, have been shown to impair wound healing in various clinical contexts [20]. Several studies have reported an increased risk of wound healing complications in patients preoperatively treated with bevacizumab for liver metastases [21,22]. Bevacizumab has also been associated with a wound healing complication rate as high as 35% in patients undergoing craniotomy for brain tumors [23].

In clinical settings, surgeries are frequently performed under time constraints, leaving insufficient time for the pharmacological effects of MTD to subside. To address this, we developed the CSI method as an intraoperative preventative strategy to minimize the risk of wound dehiscence postoperative spinal cord metastasis. This technique involves making a skin incision outside the irradiated area, thereby reducing potential complications from radiotherapy. Typically, irradiation is administered to three vertebra bodies: the affected vertebral body and one vertebra above and below it, with a margin of approximately 0.5 cm from the tumor edge. To accommodate for body movement and wound margin retraction, a preoperative skin incision was placed on the back of the vertebral body, ensuring it was at least 2.5 cm away from the vertebral body margin.

Previously reported strategies to prevent wound dehiscence include delaying surgery for 6–8 weeks after radiotherapy and employing tissue flap reconstruction. However, each approach has limitations: delayed surgery may increase the risk of neurological deterioration, while flap reconstruction requires specialized microsurgical expertise that may not be available in all surgical centers [24,25]. In contrast, CSI offers a simple, cost-effective solution that can be implemented immediately without additional resources or specialized training.

In this study, propensity score matching was used to reduce treatment selection bias when comparing CSI and MI. An analysis of the propensity-matched groups demonstrated that CSI effectively reduced the risk of postoperative wound dehiscence, highlighting its potential as a novel preventative treatment. Although we observed a case of skin ulceration in the irradiated area following the introduction of CSI, wound dehiscence was successfully prevented. These findings underscore the clinical significance of CSI as a straightforward, facility-independent procedure that effectively prevents postoperative wound dehiscence in patients with spinal metastasis surgery who have undergone preoperative irradiation.

Minimally invasive surgical techniques, such as the use of percutaneous pedicle screws, offer the advantage of inserting screws from outside the irradiation area, thereby reducing the risk of wound dehiscence. However, it is not always possible to completely avoid the irradiation zone. The increasing incidence of spinal metastases in the era of improved cancer survival rates demands more frequent surgical interventions, often in patients with a history of radiotherapy. This clinical reality increases the importance of techniques such as CSI that can reduce complications in irradiated tissues while maintaining surgical efficacy. Our research has evaluated spine surgery for spinal metastases from both medical and economic perspectives. Our goal is to standardize the procedure so that it can be performed by all spine surgeons, including facilities lacking specialized surgical equipment such as navigation systems or surgical robots. Conventional open surgery using the novel CSI method is a relatively simple and accessible technique that we believe holds significant promise in reducing complication rates, making it an effective and accessible option for spinal metastasis surgery.

This study has a few limitations. First, this was a retrospective, single-center study with a relatively small study population. Second, due to the limited number of patients in this study, we did not examine the effects of other possible risk factors for postoperative wound dehiscence, such as steroid use, diabetes mellitus, or the interval between preoperative radiation and surgery [18] in the multivariable analyses. Additionally, this study did not distinguish between individual drugs within the MTD group. The drugs associated with postoperative wound dehiscence in this study were those that affect VEGF levels, whereas other MTDs caused minimal postoperative wound dehiscence.

## 5. Conclusions

Our findings demonstrated that the CSI technique is a practical and effective method for reducing postoperative wound complications in spinal metastasis surgery. The technique showed clear clinical benefit, particularly in patients with a history of radiotherapy. It is straightforward, cost-neutral, and widely applicable, making it a valuable tool for spine surgeons treating the growing population of cancer survivors. Future studies should validate its effectiveness across broader populations and explore its application in other surgical contexts.

## Figures and Tables

**Table 1:** Demographics and baseline clinical characteristics of 107 patients with midline incision (MI; enrollment period: 2013–2018) and 92 patients with MI or curved skin incision (enrollment period: 2019–2021).

	MI, Enrolled in 2013–2018			MI/CSI, Enrolled in 2019–2021		
Characteristics	Total	Dehiscence	No Dehiscence	Total	Dehiscence	No Dehiscence
	(n = 107)	(n = 9)	(n = 98)	(n = 92)	(n = 8)	(n = 84)
Age, years (range)	69 (38–90)	69 (59–83)	69 (38–90)	71 (42–88)	70 (64–74)	71 (42–88)
Sex (male/female)	65:42	7:2	58:40	57:35	4:4	53:31
BMI, kg/m^2^ (range)	20.4 (13.8–38.3)	19.7 (15.6–23.8)	20.4 (13.8–38.3)	20.4 (14.7–33.9)	22.1 (14.9–26.0)	20.1 (14.7–33.9)
Revised Katagiri score (range)	6 (0–9)	6 (3–7)	6 (0–9)	5 (0–9)	5 (3–8)	5 (0–9)
Smoking history, n (%)	45 (42.1%)	3 (33.3%)	42 (42.9%)	31 (33.7%)	6 (75.0%)	25 (29.8%)
Preoperative radiotherapy, n (%)	3 (29.0%)	8 (88.9%)	23 (23.5%)	22 (23.9%)	4 (50.0%)	18 (21.4%)
Preoperative chemotherapy, n (%)	50 (45.9%)	7 (77.8%)	44 (44.9%)	42 (45.7%)	3 (37.5%)	39 (46.4%)
Preoperative MTD therapy, n (%)	17 (15.9%)	6 (66.7%)	11 (11.2%)	18 (19.6%)	2 (25.0%)	16 (19.0%)
**Surgical information**						
Blood loss, mL (range)	280 (0–2500)	460 (0–600)	258 (0–2500)	200 (0–1268)	312 (90–560)	190 (0–1268)
Operation time, min (range)	197 (73–450)	168 (73–450)	201 (99–373)	208 (108–424)	196 (167–254)	212 (108–424)

Data are presented as count (%) or median (range). BMI: body mass index; MTD: molecular target drug; MI: midline incision; CSI: curved skin incision.

**Table 2:** Postoperative complications in 107 patients with a midline incision, enrolled in 2013–2017, during the 6-month follow-up.

Complication	N (%)
Wound dehiscence	9 (8.4%)
Fracture	4 (3.7%)
Pneumonia	4 (3.7%)
Rod failure	3 (2.8%)
DVT	3 (2.8%)
Paralysis	3 (2.8%)
Depression	2 (1.9%)
Cerebrospinal fluid leaking	2 (1.9%)
Others	5 (4.7%)

DVT: deep vein thrombosis.

**Table 3:** Multivariable analysis of risk factors for postoperative wound dehiscence in patients with midline incision.

Risk Factor	Odds Ratio	95% Confidence Interval	*p*-Value
Age (>65 years)	1.063	0.133–8.492	0.954
Sex (male)	2.684	0.253–28.482	0.413
BMI	1.058	0.767–1.459	0.731
Revised Katagiri score	1.052	0.562–1.966	0.875
Smoking history	0.546	0.054–5.489	0.607
Preoperative radiotherapy	32.599	2.968–358.060	0.004 *
Preoperative chemotherapy	0.314	0.024–4.092	0.376
Preoperative MTD therapy	25.242	1.802–353.617	0.017 *
Blood loss	1.001	0.998–1.004	0.615
Operation time	0.997	0.986–1.009	0.668

* indicates statistically significant results. BMI: body mass index; MTD: molecular target drug.

**Table 4:** Characteristics of 92 patients with MI or CSI (enrollment period: 2019–2021).

	**Before Propensity Score Matching**			**After Propensity Score Matching**		
	**MI** **(n = 59)**	**CSI (n = 33)**	***p*-Value**	**MI** **(n = 29)**	**CSI** **(n = 29)**	***p*-Value**
Age ≥ 65 years	42 (71.2%)	24 (72.7%)	>0.999	21 (72.4%)	21 (72.4%)	>0.999
Sex (male)	34 (57.6%)	23 (69.7%)	0.273	20 (69.0%)	20 (69.0%)	>0.999
BMI, kg/m^2^	21.3 ± 3.8	21.1 ± 3.5	0.880	21.4 ± 3.8	21.2 ± 3.8	0.820
Revised Katagiri score	4.6 ± 2.2	5.2 ± 2.2	0.294	5.2 ± 2.2	5.1 ± 2.3	0.589
Smoking	18 (30.5%)	13 (39.4%)	0.491	11 (37.9%)	10 (34.5%)	0.787
Preoperative radiotherapy	11 (18.3%)	11 (33.3%)	0.132	10 (34.5%)	9 (31.0%)	>0.999
Preoperative chemotherapy	27 (45.8%)	15 (45.5%)	>0.999	14 (48.3%)	15 (58.6%)	0.599
Preoperative MTD therapy	10 (16.9%)	6 (18.2%)	>0.999	8 (27.6%)	6 (27.6%)	>0.999
Blood loss, mL	240 ± 221	354 ± 330	0.051	295 ± 255	300 ± 291	0.204
Operation time, min	206 ± 54	214 ± 64	0.518	205 ± 54	206 ± 54	0.961
Dehiscence	7 (11.9%)	1 (3.0%)		6 (20.7%)	0 (0.0%)	0.024

Data are presented as N (%) or medians. MI, midline incision; CSI, curved skin incision; BMI, body mass index; MTD, molecular targeted drug.

## Data Availability

The data presented in this study are available upon request from the corresponding author. The data are not publicly available due to privacy restrictions.

## Supplementary Materials

The following supporting information can be downloaded at https://www.mdpi.com/article/10.3390/cancers17121973/s1, Table S1: Names of drugs and postoperative wound dehiscence in 17 cases with MI where preoperative MTD was administered (enrollment period: 2013–2017); Table S2: Names of drugs and postoperative wound dehiscence in 16 patients with MI or CSI where preoperative MTD was administered (enrollment period: 2019–2021).

## Abbreviations

The following abbreviations are used in this manuscript:

CSI	curved skin incision
MI	midline incision
SSM	symptomatic spinal metastases
PS	performance status
ADL	activities of daily living
QOL	quality of life
BMI	body mass index
MTD	molecular target drug
VEGF	vascular endothelial growth factor

## References

[1] Coleman R.E. (2001). Metastatic bone disease: Clinical features, pathophysiology and treatment strategies. Cancer Treat. Rev..

[2] Lee G.H., Kim H.I., Park J.K., Choi Y.S., Han J.H., Lim S.D. (2024). Contemporary trends in the incidence and timing of spinal metastases. J. Neurosurg. Spine.

[3] Long N., Woodlock D., D’Agostino R., Nguyen G., Gangai N., Sevilimedu V., Do R.K.G. (2025). Incidence and prevalence of bone metastases in different solid tumors determined by natural language processing of CT reports. Cancers.

[4] Kakutani K., Kanda Y., Yurube T., Maeno K., Takada T., Kurakawa T., Takeoka Y., Miyazaki S., Hoshino Y., Nishida K. (2023). The identification of risk factors for symptomatic spinal metastasis onset: A prospective cohort study of 128 asymptomatic spinal metastasis patients. Cancers.

[5] Lutz S., Berk L., Chang E., Chow E., Hahn C., Hoskin P., Howell D., Konski A., Kachnic L., Lo S. (2011). Palliative radiotherapy for bone metastases: An ASTRO evidence-based guideline. Int. J. Radiat. Oncol. Biol. Phys..

[6] Patchell R.A., Tibbs P.A., Regine W.F., Payne R., Saris S., Kryscio R.J., Mohiuddin M., Young B. (2005). Direct decompressive surgical resection in the treatment of spinal cord compression caused by metastatic cancer: A randomised trial. Lancet.

[7] Choi D., Crockard A., Bunger C., Harms J., Kawahara N., Mazel C., Melcher R., Tomita K., Global Spine Tumor Study Group (2010). Review of metastatic spine tumour classification and indications for surgery: The consensus statement of the Global Spine Tumour Study Group. Eur. Spine J..

[8] Kanda Y., Kakutani K., Sakai Y., Miyazaki K., Matsuo T., Yurube T., Takeoka Y., Ohnishi H., Ryu M., Kumagai N. (2023). Clinical characteristics and surgical outcomes of metastatic spine tumors in the very elderly: A prospective cohort study in a super-aged society. J. Clin. Med..

[9] Fomchenko E.I., Bayley J.C., Alvarez-Breckenridge C., Rhines L.D., Tatsui C.E. (2022). Spinal metastases and the evolving role of molecular targeted therapy, chemotherapy, and immunotherapy. Neurospine.

[10] Finkelstein J.A., Zaveri G., Wai E., Vidmar M., Kreder H., Chow E. (2003). A population-based study of surgery for spinal metastases: Survival rates and complications. J. Bone Jt. Surg. Br..

[11] Matsuo T., Kanda Y., Sakai Y., Yurube T., Takeoka Y., Miyazaki K., Ohnishi H., Ryu M., Kumagai N., Kuroshima K. (2024). Modified frailty index as a novel predictor for the incidence and severity of postoperative complications after spinal metastases surgery: A prospective cohort study. Bone Jt. J..

[12] Zhu X., Li J., Peng S., Wang J., Zhang L. (2020). Radiation-induced skin injury: Pathogenesis, treatment, and management. Aging.

[13] Lau D., Chou D. (2015). Posterior thoracic corpectomy with cage reconstruction for metastatic spinal tumors: Comparing the mini-open approach to the open approach. J. Neurosurg. Spine.

[14] Rao P.J., Thayaparan G.K., Fairhall J.M., Mobbs R.J. (2014). Minimally invasive percutaneous fixation techniques for metastatic spinal disease. Orthop. Surg..

[15] Borrelli M.R., Shen A.H., Lee G.K., Momeni A., Longaker M.T., Wan D.C. (2019). Radiation-induced skin fibrosis: Pathogenesis, current treatment options, and emerging therapeutics. Ann. Plast Surg..

[16] Katagiri H., Okada R., Takagi T., Takahashi M., Murata H., Harada H., Nishimura T., Asakura H., Ogawa H. (2014). New prognostic factors and scoring system for patients with skeletal metastasis. Cancer Med..

[17] Carl H.M., Ahmed A.K., Abu-Bonsrah N., De la Garza Ramos R., Sankey E.W., Pennington Z., Bydon A., Witham T.F., Wolinsky J.P., Gokaslan Z.L. (2018). Risk factors for wound-related reoperations in patients with metastatic spine tumor. J. Neurosurg. Spine.

[18] Sugita S., Hozumi T., Yamakawa K., Goto T., Kondo T. (2016). Risk factors for surgical site infection after posterior fixation surgery and intraoperative radiotherapy for spinal metastases. Eur. Spine J..

[19] Ghogawala Z., Mansfield F.L., Borges L.F. (2001). Spinal radiation before surgical stabilization and instrumentation: A study of wound complications. J. Neurosurg..

[20] Bose D., Meric-Bernstam F., Hofstetter W., Reardon D.A., Flaherty K.T., Ellis L.M. (2010). Vascular endothelial growth factor targeted therapy in the perioperative setting: Implications for patient care. Lancet Oncol..

[21] Scappaticci F.A., Fehrenbacher L., Cartwright T., Hainsworth J.D., Heim W., Berlin J., Kabbinavar F.F., Hurwitz H.I., Novotny W.F., Malik I. (2005). Surgical wound healing complications in metastatic colorectal cancer patients treated with bevacizumab. J. Surg. Oncol..

[22] Mahfud M., Breitenstein S., El-Badry A.M., Puhan M.A., Slankamenac K., Graf R., Clavien P.A. (2010). Impact of preoperative bevacizumab on complications after resection of colorectal liver metastases: Case-matched control study. World J. Surg..

[23] Ladha H., Pawar T., Gilbert M.R., Sloan A.E., Weil R.J. (2011). Impact of bevacizumab chemotherapy on craniotomy wound healing in patients with brain tumors. J. Neurosurg..

[24] Chieng L.O., Hubbard Z., Salgado C.J., Levi A.D., Chim H. (2015). Reconstruction of open wounds as a complication of spinal surgery with flaps: A systematic review. Neurosurg. Focus.

[25] Itshayek E., Yamada J., Bilsky M., Schmidt M., Shaffrey C., Gerszten P., Polly D., Gokaslan Z., Varga P.P., Fisher C.G. (2010). Timing of surgery and radiotherapy in the management of metastatic spine disease: A systematic review. Int. J. Oncol..

