# Supplementary material for: Risk Factors for Wound Dehiscence After Spinal Metastasis Surgery and a New Approach to Prevention—Curved Skin Incision
Source: Cancers (Basel). 2025 Jun 13;17(12):1973. doi: 10.3390/cancers17121973 (PMC12191379; doi:10.3390/cancers17121973)
Supplement: Supplementary file 1 [file cancers-17-01973-s001.zip › cancers-3646158-supplementary.pdf]

Supplementary Material

Table S1. Names of drugs and postoperative wound dehiscence in 17 cases with MI where pre-operative MTD was administered (enrollment period: 2013-2017).

MTD	Total	No. of Wound dehiscence	Rate (%)
Bevacizumab	5	2	40.0%
Sunitinib	2	1	50.0%
Sorafenib	1	1	100.0%
Sunitinib/ Sorafenib	1	1	100.0%
Sunitinib/ Temsirolimus	1	1	100.0%
Bortezomib	2	0	0.0%
Afatinib	1	0	0.0%
Gefitinib	1	0	0.0%
Pembrolizumab	1	0	0.0%
Temsirolimus	1	0	0.0%
Trametinib	1	0	0.0%
Total	17	6	35.3%

MTD: molecular target drug

Table S2. Names of drugs and postoperative wound dehiscence in 16 patients with MI or CSI where pre-operative MTD was administered (enrollment period: 2019-2021).

MTD	Total	No.of Wound dehiscence	Rate (%)
Pembrolizumab	6	1	16.7%
Nivolumab	2	0	0.0%
Sorafenib	1	1	100.0%
Bevacizumab	1	0	0.0%
Bevacizumab, Cetuximab	1	0	0.0%
Sunitinib, Axitinib	1	0	0.0%
Pazopanib	1	0	0.0%
Axitinib	1	0	0.0%
Bortezomib	1	0	0.0%
Nivolumab, Ipilimumab	1	0	0.0%
Total	16	2	12.5%

MTD: molecular target drug
